# In HCV-related liver cirrhosis, local pulse wave velocity increases and in decompensated patients correlates with poorer survival

**DOI:** 10.1371/journal.pone.0212770

**Published:** 2019-03-19

**Authors:** Chien-Hao Huang, Lung-Sheng Wu, Wen-Juei Jeng, Yu-Fu Cheng, Yu-Shien Ko, I-Shyan Sheen, Chun-Yen Lin

**Affiliations:** 1 Division of Hepatology, Department of Gastroenterology and Hepatology, Chang-Gung Memorial Hospital, Linkou Medical Center, Taoyuan, Taiwan; 2 College of Medicine, Chang-Gung University, Taoyuan, Taiwan; 3 Graduate Institute of Clinical Medical Sciences, College of Medicine, Chang-Gung University, Taoyuan, Taiwan; 4 Department of Cardiology, Chang-Gung Memorial Hospital, Linkou Medical Center, Taoyuan, Taiwan; 5 School of Traditional Chinese Medicine, College of Medicine, Chang-Gung University, Taoyuan, Taiwan; 6 Department of Internal medicine, Chang-Gung Memorial Hospital, Chiayi Branch, Chiayi, Taiwan; University of Palermo, ITALY

## Abstract

**Background:**

Cirrhotic cardiomyopathy (CCM) refers to cardiac dysfunction in patients with liver cirrhosis, in the absence of other known cardiac disease.

**Methods:**

Control group and patients diagnosed of liver cirrhosis without known cardiac disease or hepatocellular carcinoma were enrolled for this clinical observation study. Patients with diabetes mellitus, hypertension were excluded. Absolute global longitudinal strain, one-point carotid pulse wave velocity (one-point PWV) and various parameters were measured in resting status.

**Results:**

There were 29 participants in the control group and 80 patients in the liver cirrhosis group. 27.8% of cirrhotic patients presented with normal systolic but abnormal diastolic functions and QTc prolongation that were compatible with CCM. 34.2% of cirrhotic patients presented with diastolic dysfunction in resting state comparing to 24.1% in control group. Systolic functions did not show conspicuous difference between cirrhosis and control group nor between compensated and decompensated cirrhosis, neither. Furthermore, one-point PWV was significantly higher in liver cirrhosis than in control group and higher in CCM than in non-CCM patients. One-point PWV predicted CCM and diastolic dysfunction in cirrhosis. Most importantly, its value > 1370cm/s predicted overall mortalities in decompensated cirrhosis (multivariable Cox analysis OR = 6.941) in addition to CTP score specifically in HCV related cirrhotic patients (AUC = 0.817).

**Conclusions:**

In patients with cirrhosis, 27.8% were diagnosed with CCM by resting cardiovascular parameters. One-point PWV increased in CCM, correlated with diastolic dysfunction. It also correlated with overall mortality in patients with hepatitis C virus (HCV) related decompensated cirrhosis. Further study may be needed to confirm its capability for assessing CV and mortality risks in HCV related decompensated cirrhotic patients.

## Introduction

The term cirrhotic cardiomyopathy (CCM) is used to describe cirrhotic patient with normal-to-elevated cardiac output and contractility at rest but a blunted response to pharmacologic, physiologic, or pathologic stress[1]. Reduced peripheral resistance, neuroendocrine dysfunction, and electrophysiological abnormalities is an independent contributor to cardiac dysfunction[2].

Because of the presence of substantial peripheral vasodilation, cirrhotic patients are less likely to develop severe or overt heart failure[2]. Therefore, when cardiovascular stress is absent, modest subclinical abnormalities in diastolic or systolic function do not need treatment [2]. However, situations that involve substantial cardiac stress, such as sepsis, surgery, or transjugular intrahepatic portosystemic shunt insertion, may reveal the limited ventricular reserve and cause severe heart failure[2] or death after liver transplantation[3]. It is therefore important to investigate latent heart failure at rest or before stress and to identify cardiac factors associated with mortality.

Newer echocardiographic modalities such as tissue Doppler echocardiography enable detection of myocardial function on the long axis and provide additional information on myocardial function[4, 5]. Speckle-tracking echocardiography is an even newer method for detecting subclinical left ventricular dysfunction in latent heart failure[6, 7]. It measures velocities relative to adjacent velocities. Speckle-tracking–derived strain may overcome some tissue Doppler limitations such as image artifacts and dependence on insonation angle, thereby improving reproducibility[8, 9]. However, existing evidence does not clearly indicate if cirrhosis-related ventricular function increases or decreases in the resting state[9–11].

Pulse wave velocity (PWV) is considered one of the most important clinical parameters for evaluate the CV risk, vascular adaptation, and therapeutic efficacy[12]. Commercial devices dedicate to PWV measurements make a regional assessment, i.e., the PWV measured between two vessels. However the advantages of a local measurement like one-point carotid PWV (one-point PWV) is evident especially in the detection of early stage of atherosclerosis disease[13].

Only a few studies have investigated the prognosis for liver cirrhosis-related systolic, diastolic dysfunction, and change in vascular resistance before opportune liver transplantation[14]. We analyzed various cardiovascular parameters including CCM in liver cirrhosis and relevant prognostic factors in a region where viral hepatitis is more endemic.

## Materials and methods

### Study population

This research was a clinical observation study and its protocol conformed to the ethical guidelines of the 1975 Declaration of Helsinki and was approved by the ethical committees of Chang Gung Memorial Hospital (104-2796B: 2015-5-29). All the enrolled participants had read carefully then signed the informed consent form.

Controls group participants without any apparent diseases or infections were recruited from health check-up center in Chang Gung Memorial Hospital.

Patients with liver cirrhosis were recruited from outpatient clinic or liver wards of the Chang Gung Memorial Hospital. The inclusion criteria included (i) liver cirrhosis, which was based on a histopathological diagnosis or a combination of compatible clinical features, laboratory data, and imaging findings. (ii) no evidence of remarkable HCC or other metastatic liver tumor; and (iii) no β-blocker nor other vasoactive drugs in use within 2 days prior to study entry, (iv) ages between 35 to 65 years old.

Exclusion criteria include: (i) DM (diabetes mellitus) at entry (ii) HTN (hypertension) /Shock (iii) ESRD(end-stage renal disease) (iv) Heart disease (v) Severe alcoholic hepatitis or acute liver failure.

Patients were followed at least 1 year after enrolling or until orthotopic liver transplantation (OLT)/death for their outcome analysis. Patients were managed according to current APASL guidelines.

### Study protocol

Patients had their blood tests when meeting medical needs during their regular OPD visits or hospitalization courses without additional blood drawings. Patients took beta-blocker according to the Baveno VI guideline[15] and was hold for 2 days before the examination if no absolute contraindication. Overnight fasting cardiac and peripheral vascular examinations including 2D color Doppler echocardiography, speckle tracking strain software and peripheral vascular tests including Doppler, pulse volume/cuff pressure recorders and phleborheography (PRG) were performed by an experienced cardiologist as clinical practice purposes to exclude cardiovascular lesions such as heart failure or vascular thrombosis, without charging either to patients or national health insurance.

The diagnosis of CCM was based on the expert consensus committee at the World Congress of Gastroenterology in Montreal except for the systolic function part, since in our study systolic function was not measured in response to physiologic or pharmacologic strain[16].

Concerning global longitudinal strain (GLS), the endocardial borders were traced in the end-systolic frame of the 2D images from the 3 apical views. Speckles were tracked frame-by-frame throughout the LV wall during the cardiac cycle (EchoPAC Dimension 06; GE Healthcare Corp.). GLS was calculated as the mean strain of 18 segments, as shown in S1 Fig. For simplicity and avoiding confusion, GLS had been converted into absolute values to depict comparisons as recommended by the European Association of Cardiovascular Imaging (EACVI)/ASE/Industry Task Force to standardize deformation imaging[17]. An absolute GLS value of ≤18% was defined as abnormal in our study[18]. The diagnosis of diastolic dysfunction was also in accordance with Montreal consensus criteria mentioned above, which was E/A ratio < 1.0[16].

Pulse wave velocity (PWV) is a non-invasive assessment of arterial stiffness. PWV is defined as the pulse wave travel speed throughout the aorta. A PWV increase as the aorta becomes stiffer, which is a factor that determines the development of cardiovascular complications. Previously we used brachial-ankle pulse wave velocity to measure arterial stiffness with an automated device (Colin VP-1000, Omron, Kyoto, Japan), as described previously[19]. In this study, we further advanced to measure one-point common carotid artery pulse wave velocity by taking advantage of the high-definition echo-tracking system (ProSound Alpha10; Aloka, Tokyo, Japan) to simulate local PWV in order to more accurately reflect pathophysiologic condition of the aorta, which had been explicitly mentioned in Olga Vriz et al., study[20].

Arterial resistances, including carotid arterial compliance (AC) and carotid augmentation index (AI), were measured by vascular ultrasonography, as shown in S2 Fig. The corrected QT interval (QTc) was calculated by using the Fridericia correction formula[21]. QTc cutoff point was defined as 440ms in this study, as previous studies found an increased risk of sudden cardiac death beyond the value[22–24].

Cirrhotic patients were subdivided into compensated (CTP score ≤A6) and decompensated group (CTP score ≥B7) groups[25] due to a dramatic increase of life-threatening complications and mortalities[26, 27]

### Statistical analysis

Statistics were performed using SPSS software (SPSS Inc., Chicago, IL, USA). Statistical methods of this study were reviewed by Center for Big Data Analytics and Statistics of Chang Gung Memorial Hospital. Concerning continuous variables that are **Gaussian distributed**, they are expressed as mean ± SD and the independent t-test was used for comparisons between two groups, while one-way ANOVA was used for comparisons among three groups. When p value <0.05, post hoc analysis was performed to evaluate between which groups there was a statistical significance. As for continuous variables that are not Gaussian distributed, they are expressed as median (IQR) and the Mann-Whitney U test was used for comparisons between two groups, while Kruskal Wallis test was used for comparisons among three groups. Categorical variables are expressed as frequencies or percentages and calculated by Chi-square test first while Fisher’s exact test was performed instead when more than 20% of the cells have expected frequencies < 5. As for correlation evaluations, Pearson product moment method was used for two continuous variables while Spearman rank-order correlation was used for non-continuous variables. Multivariable analyses were performed by either multiple linear or logistic regression analysis. The Kaplan–Meier (K-M) and Log-rank test were used for univariable survival analysis while Cox regression model was used for multivariable survival analysis. As for mortality prediction by one-point PWV, the optimal cut-off point was generated first by the Youden’s index method (the highest value of sensitivity+specificity-1), and then the area under the receiver operating characteristic (AUROC) curve or AUC was calculated accordingly to assess the predictive ability. A p value of < 0.05 was considered statistically significant.

## Results

### Demographics between control group vs. liver cirrhosis, compensated vs. decompensated cirrhosis and among different etiologies of cirrhotic patients: Highest TG in alcoholic cirrhosis

In total, 29 control group participants (19 men, 10 women; mean age 48±8years) and 80 cirrhotic patients (64 men, 16 women; mean age 51±8years) who met the inclusion and exclusion criteria were enrolled (Table 1). The mean duration of follow-up for cirrhotic patients was 561.56±43.24 days. There were no significant differences between control group and liver cirrhosis in gender, age and serum creatinine (Table 1). Serum total cholesterol (T-Chol) levels and TG were higher in control group than in liver cirrhosis (Table 1), which corresponded to other study[28] that the concentration of cholesterol and TG in liver cirrhosis were significantly decreased in comparison with the control group. Serum AST, ALT and bilirubin total were significantly higher in liver cirrhosis than in control group while serum albumin was significantly lower in liver cirrhosis than in control group (Table 1).

**Table 1:** Demographic characteristic of normal controls and patients with liver cirrhosis (compensated vs. decompensated).

Parameters*	Control group (n = 29)	Cirrhosis (n = 80)	P value	Liver Cirrhosis Compensated Decompensated (n = 31) (n = 49)		P value
**Male, n (%)**	19 (65.5)	64(80.0)	0.117	24(77.4)	40(81.6)	0.646
**Age (years)**	49.0(43.0–52.5)	48.5(45.0–59.0)	0.227	54.0(47.0–62.0)	48.0(43.5–54.5)	**0.037**
**Cirrhosis Etiologies**			N/A			0.102
**Alcohol, n (%)**		28(25.7)		7(22.6)	21(42.9)	
**HBV, n (%)**		22(20.2)		12(38.7)	10(20.4)	
**HCV, n (%)**		30(27.5)		12(38.7)	18(36.7)	
**AST (U/L)**	20.0 (18.0–23.0)	71.5(39.0–101.8)	**<0.001**	39.0(30.0–72.0)	77.0(48.5–108.5)	**<0.001**
**ALT (U/L)**	18.0(15.0–27.0)	32.0(19.3–52.5)	0.011	28.0(20.0–41.0)	33.0(16.0–64.0)	0.628
**Cr (mg/dL)**	0.9(0.7–1.1)	0.8(0.5–1.0)	0.065	0.7(0.5–0.9)	0.8(0.6–1.1)	**0.121**
**Bilirubin T (mg/dL)**	0.9(0.8–1.2)	1.8(1.0–3.7)	<0.001	0.9(0.7–1.4)	2.7(1.8–7.8)	**<0.001**
**Albumin (g/dL)**	4.8(4.6–4.9)	2.9(2.4–3.3)	<0.001	3.7(3.1–4.5)	2.6(2.2–3.0)	**<0.001**
**INR (international normalized ratio)**	1.0(1.0–1.0)	1.4(1.2–1.7)	<0.001	1.2(1.0–1.4)	1.6(1.4–2.0)	**<0.001**
**Ascites, yes, n (%)**	N/A	40(50.0)	N/A	0	40(81.6)	**<0.001**
**HE, yes, n (%)**	N/A	25 (31.3)	N/A	0	25(51.0)	**<0.001**
**Na (mEq/L)**	N/A	137.0±4.4	N/A	139.0(136.8–141.3)	137.0(135.0–139.0)	0.031
**K (mmol/L)**	N/A	3.8±0.6	N/A	3.8±0.5	3.9±0.6	0.841
**T-Chol (mg/dL)**	196.0±27.0	146.7±40.8	**<0.001**	144.7±35.6	149.1±47.6	0.760
**TG (mg/dL)**	129.5(116.0–163.0)	82.0(64.0–129.5)	**0.032**	78.0(64.0–120.0)	108.5(63.8–130.8)	0.563
**Systolic function**						
**Ejection Fraction (EF) (%)**	69.1±7.1	70.0±7.3	0.572	68.4±6.6	71.0±7.5	0.118
**Absolute GLS**	20.2(19.1–23.0)	21.5(20.4–22.4)	0.108	21.3±2.4	21.6±2.1	0.506
**Diastolic dysfunction,n (%)**^#^	7 (24.1)	27(34.2)	0.319	12 (40.0)	15 (30.6)	0.393
**EPS: QTc (ms)**	419.0(404.0–428.5)	453.5(430.5–483.5)	**<0.001**	440.0(425.0–466.5)	464.0(434.0–502.0)	**0.028**
**Cardiac output (L/min)**	4.9(4.3–5.5)	6.1(4.6–8.0)	**0.001**	5.1(4.3–6.3)	6.7(5.3–8.9)	**<0.001**
**Carotid arterial compliance (mm2/kPa)**	0.7(0.7–0.7)	0.9(0.7–1.1	**0.049**	0.8(0.6–0.9)	0.9(0.7–1.2)	**0.009**
**PWV one-point (cm/s)**	1239.0±97.5	1503.7±406.4	**<0.001**	1616.1±368.2	1442.1±416.1	0.052
**Left Atriam diameter (mm)**	37.0(33.5–39.0)	38.0(33.0–41.0)	0.404	34.4±4.6	39.5±5.1	**<0.001**
**Left ventricular diastolic diameter (mm)**	47.7±3.5	48.8±5.2	0.363	46.2±3.8	50.4±5.3	**<0.001**
**CCM, n (%)**	0 (0)	22(27.8)	0.001	8(26.7)	14(28.6)	0.855
**Mortality rate n (%)**	N/A	18(23.1)	N/A	3(10.0)	15(31.2)	**0.030**

* Continuous variables that are Gaussian distributed are expressed as mean ± SD; non- Gaussian distributed are expressed as median (25th percentile~75th percentile IQR)

^#^ The diagnosis of diastolic dysfunction was also in accordance with Montreal consensus criteria

HBV: hepatitis B virus; HCV: hepatitis C virus; Absolute GLS: Absolute global longitudinal strain; EPS: Eelectrophysiological parameters; QTc: corrected QT interval; CO: Cardiac output; AC: Carotid arterial compliance; PWV: Pulse wave velocity; CCM: Cirrhotic cardiomyopathy

Furthermore, cirrhotic patients were subdivided into compensated (CTP score ≤A6, 31 patients) and decompensated group (CTP score ≥B7: 22 patients in the CTP-B, and 27patients in the CTP-C) [25] due to dramatic increase of life-threatening complications and mortalities[26, 27]. There were no significant differences between compensated (compensated) and decompensated (decompensated) liver cirrhosis group in gender, etiologies of liver cirrhosis, ALT, Cr, K, T-Chol and TG (Table 1). Importantly, several clinical parameters including age, serum AST and serum Na were significantly different between compensated and decompensated cirrhosis group (Table 1). In decompensated cirrhosis, there were 10 patients (20.4%) who had mild ascites, 14 patients (28.6%) had moderate ascites, and 16 patients (32.7%) had severe ascites; 25 patients (51.0%) had hepatic encephalopathy.

Among different etiologies of liver cirrhosis patients, no significant differences were found in gender, MELD score, AST, serum creatinine and T-chol (Table 2). Age was smallest in alcoholic cirrhosis and there was a significant difference between alcoholic and HCV related liver cirrhosis by pairwise comparisons (p = 0.026). ALT were highest in HCV related liver cirrhosis and lowest in alcoholic liver cirrhosis while there were significance between them (p = 0.006) and hepatitis B virus (HBV) related vs. alcoholic liver cirrhosis (p = 0.042). TG was highest in alcohol and lowest in HBV related liver cirrhosis and there was a significant difference between them (p = 0.028) (Table 2).

**Table 2:** Demographic characteristics of control group and different etiologies of liver cirrhosis.

	Liver Cirrhosis			
Parameters	Alcohol (n = 28)	HBV (n = 22)	HCV (n = 30)	P value
**Male, n (%)**	25 (89.3)	16 (72.7)	23 (76.7)	0.295
**Age, mean±SD (years)**	45.5(42.0–54.8)	48.0(47.0–60.5)	53.5(48.0–59.5)	**0.032**
**MELD score**	15.5(11.5–22.5)	10.0(8.0–14.0)	14.0(8.8–21.0)	0.135
**AST (U/L)**	73.0(37.5–104.3)	63.5(38.0–87.0)	66.0(38.3–140.3)	0.570
**ALT (U/L)**	21.5(13.0–34.8)	35.0(25.3–55.0)	37.5(23.0–93.0)	**0.005**
**Cr (mg/dL)**	0.6(0.4–1.0)	0.8(0.6–1.1)	0.8(0.5–0.9)	0.261
**T-Cholesterol (mg/dL)**	138.3±34.2	157.4±34.7	143.8±51.1	0.551
**TG (mg/dL)**	120.0(77.0–132.0)	64.0(57.0–78.0)	82.5(65.0–123.0)	**0.034**
**Ejection Fraction (%)**	70.3±8.1	70.1±6.7	69.6±7.0	0.945
**Absolute GLS**	20.6±2.3	22.4±2.5	21.9±1.6	**0.034**
**Diastolic dysfunction (%)**	14 (51.9)*	5 (22.7)	8 (26.7)	0.055
**QTc (ms)**	471.0(450.0–502.0)	442.0(429.5–475.5)	441.0(422.3–468.5)	**0.006**
**Cardiac output (L/min)**	6.9±1.7	6.1±2.5	6.0±2.0	0.264
**Carotid arterial compliance (mm2/kPa)**	0.9(0.7–1.2)	0.9(0.6–1.2)	0.8(0.6–1.0)	0.267
**One point PWV (cm/s)**	1538.0±409.6	1419.1±340.9	1534.1±451.1	0.593
**Left Atrium diameter (mm)**	39.9±4.7	37.2±6.4	35.5±4.7	**0.008**
**Left ventricular diastolic diameter (mm)**	50.0±5.2	48.5±5.4	47.9±4.8	0.270
**CCM, n (%)**	11 (40.7) *	4 (18.2)	7 (23.3)	0.169
**Mortality rate (%)**	33.3	9.1	24.1	0.132

* One patient did not complete diastolic function survey, thus cannot judge if CCM.

HBV: hepatitis B virus; HCV: hepatitis C virus; T-cholesterol: Total cholesterol; TG: Triglyceride; QTc: corrected QT interval

### Cardiac function and electrophysiological parameters: Lowest absolute GLS and longest QTc in alcoholic liver cirrhosis

Systolic function, including EF (Control group: 69±7%; liver cirrhosis: 70±7%; p = 0.572) and absolute GLS (control group: 21±3%; liver cirrhosis: 22±2%, p = 0.108), did not significantly differ between control group and liver cirrhosis in the resting state without physiologic or pharmacologic challenge (Table 1). Concerning diastolic function, the percentages of participants with diastolic dysfunction did not significantly differ between control group and liver cirrhosis (control group: 24.1% [7/29]; liver cirrhosis: 34.2% [27/79], p = 0.319) (Table 1). In electrophysiological parameters (EPS), QTc significantly increased in liver cirrhosis compared to in control group (control group: 415.5±18.7 ms; liver cirrhosis: 461.1±46.1 ms; p<0.001) (Table 1). The percentage of patients with QTc prolongation (QTc≥440ms as mentioned in the Material and method section) was higher in cirrhotic patients than in control group (63% vs. 6.9%, p< 0.001).

Moreover, the cardiac functions including systolic and diastolic functions did not present a significant difference between the compensated and decompensated cirrhosis group. However, the QTc once again significantly increased in the decompensated cirrhosis group comparing to QTc in compensated cirrhosis group (decompensated: 464.0(434.0–502.0) ms; compensated: 440.0(425.0–466.5) ms; p = 0.028).

In addition, among different etiologies of liver cirrhosis, alcoholic cirrhosis had significantly the lowest absolute GLS value (vs. HBV, p = 0.049) although their EF and diastolic dysfunction percentage did not show prominent difference (Table 2). On the contrary, alcoholic liver cirrhosis had the longest QTc among them and there was a significant difference between alcoholic vs. HCV related liver cirrhosis (p = 0.007) (Table 2).

### Systemic hemodynamic status and cardiac chamber size: One-point PWV was significantly higher in patients with liver cirrhosis than control group

CO was significantly higher in liver cirrhosis patients than in control group (CO: 6.4±2.0 vs. 4.9±0.9 L/min, p<0.001). AC as peripheral resistance was also conspicuously higher in cirrhotic patients than in control group: 0.9±0.4 vs. 0.7±0.1 mm2/kPa, p = 0.020) (Table 1).

In measurement of one-point PWV (One-point PWV) for arterial stiffness as shown in S2 Fig, values prominently increased from control group to liver cirrhosis (control group: 1239.00±97.52 cm/s, cirrhosis: 1503.7 ± 406.4 cm/s; p<0.001) (Table 1). However, these one-point PWV had no significant imparity either between compensated and decompensated cirrhosis (Table 1) or among different cirrhotic etiologies (alcohol: 1538.0±409.6 cm/s, HBV: 1419.1±340.9 cm/s, HCV: 1534.1±451.1 cm/s, p = 0.539, Table 2).

In left atrial (LA) diameter and left ventricular diastolic diameter (LVEDD) of cardiac chamber sizes, no significant difference was noted between control group and liver cirrhosis (Table 1). They increased from compensated to decompensated cirrhosis (LA: p<0.001; LVEDD: p<0.001) and the longest LA diameters were in alcoholic liver cirrhosis (Table 2).

### CCM percentage in liver cirrhosis, compensated vs. decompensated cirrhosis and among different etiologies of cirrhotic patients: One-point PWV increased in CCM patients and predicted CCM in liver cirrhosis

In these liver cirrhotic patients, 27.8% met the criteria of CCM[16] (Table 1). The CCM percentage did not significantly differ either between the compensated and decompensated cirrhosis (26.7% vs. 28.6%, p = 0.855) (Table 1) or among different etiologies of cirrhotic patients (Table 2).

Furthermore, the one-point PWV of cirrhotic patients with CCM were higher than that of patients without CCM (1766.7±523.6 vs. 1414.8±311.00 cm/s, p = 0.009) (Table 3). The gender, age and MELD score did not show discrepancy between CCM vs. non-CCM (Table 3). By logistic regression, one-point PWV predicted CCM or not (OR 1.002, 95% CI 1.001–1.004, P = 0.004).

**Table 3:** Demographic characteristics of CCM and non-CCM cirrhotic patients.

	Liver Cirrhosis		
Parameters	CCM (n = 22)	Non-CCM (n = 57)	P value
**Male, n (%)**	17(77.3)	46 (80.7)	0.734
**Age, mean±SD (years)**	54.9±10.4	50.0±7.9	0.057
**MELD score**	15.9±8.3	15.3±7.9	0.763
**One-point PWV (cm/s)**	1766.7±523.6	1414.8±311.0	**0.009**

CCM: Cirrhotic cardiomyopathy;

### One-point PWV correlated and predicted diastolic dysfunction in liver cirrhosis, and was associated with overall mortalities in patients with decompensated cirrhosis

In addition to predict CCM in liver cirrhosis, one-point PWV also correlated with diastolic dysfunction (Spearman’s r = 0.460, p<0.001, S3 Fig). It was also able to predict it (OR 1.003, 95% CI 1.001–1.005, P<0.001) by univariable logistic regression analysis. It did not correlate or predict systolic dysfunction though (Sr = 0.075, P = 0.517).

To clarify the relationship between one-point PWV and mortalities, the cause of death was analyzed initially. 15 of the 78 patients died during follow-up of 561.56±43.24 days. The causes of death were divided into ① sepsis 26.6% (4/15) ② GI bleeding 26.6% (4/15) ③ Multi-organ failure 46.8%(7/15) related. Of interest, 5 of these patients (5/15 = 33.3%) also developed a major cardiovascular event (MACE) (one ACS, two stroke, one peripheral vascular occlusive disease and one ventricular tachycardia event) during follow-ups, which might contributed to mortalities. In addition, all the 5 patients with MACE were in decompensated status and 4 of these patients (80%) had one-point PWV> 1370cm/s. The MACE correlated with death in cirrhotic patients (Spearman’s r = 0.229, p = 0.043).

### One-point PWV predicted mortalities only in patients with decompensated cirrhosis

By univariable Cox regression analysis, one-point PWV predicted overall mortalities in liver cirrhosis (OR 1.001, 95% CI 1.000–1.002, P = 0.025). Subgroup analysis revealed one-point PWV predicted mortalities only in decompensated cirrhosis (OR 1.001, 95% CI 1.001–1.002, P = 0.001) but not in compensated cirrhosis (OR 0.999, 95% CI 0.995–1.003, P = 0.513).

### One-point PWV>1370 cm/s predicted mortalities only in patients with HCV related decompensated cirrhosis

**Fig 1:**
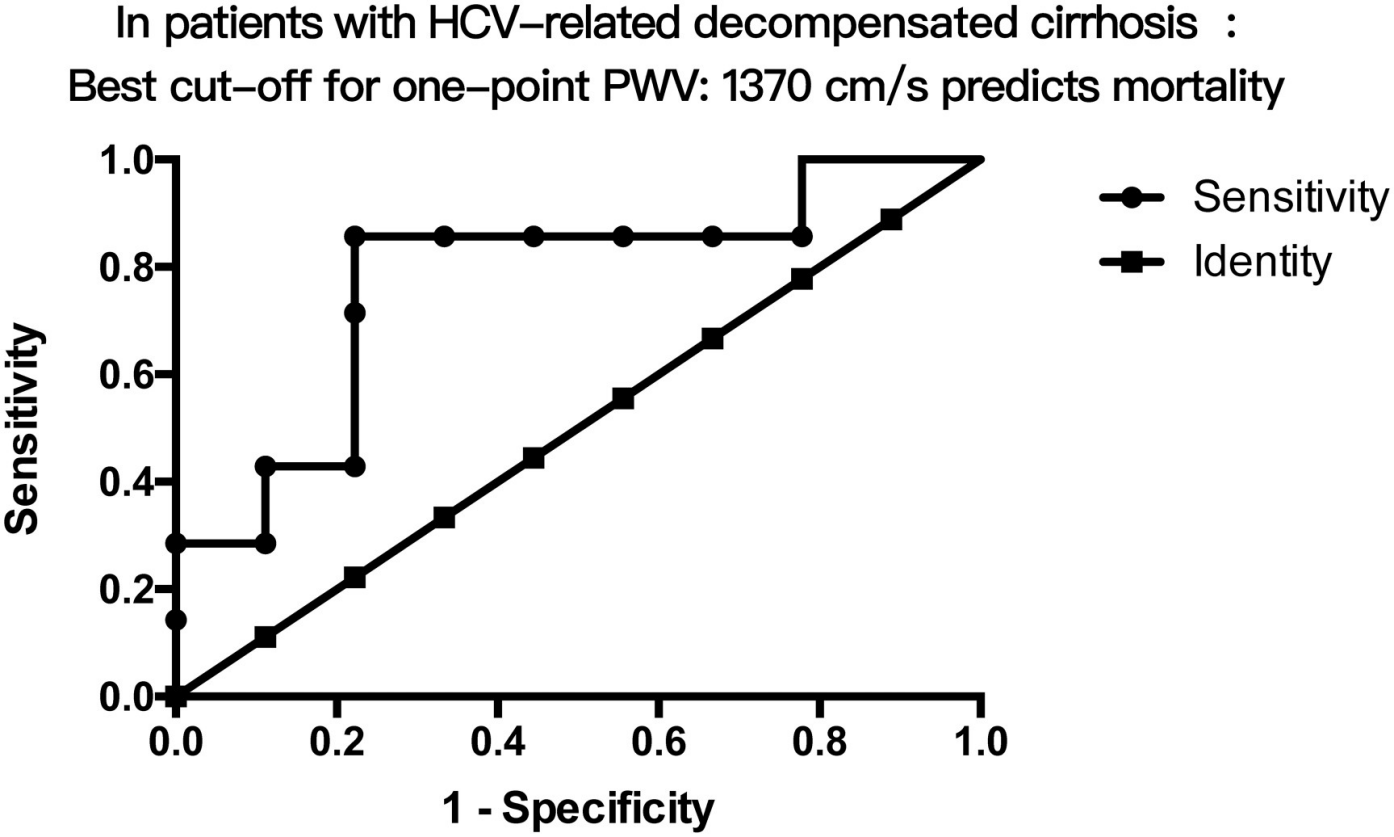
The AUC of one-point PWV in predicting mortalities in patients with HCV related decompensated cirrhosis. By Youden’s index to determine the best cut-off value (highest value of sensitivity+specificity-1), **one-point** of PWV> 1370 cm/s was the best cut-off value in predicting mortalities for HCV related decompensated cirrhosis (AUROC = 0.813, p = 0.034).

**Fig 2:**
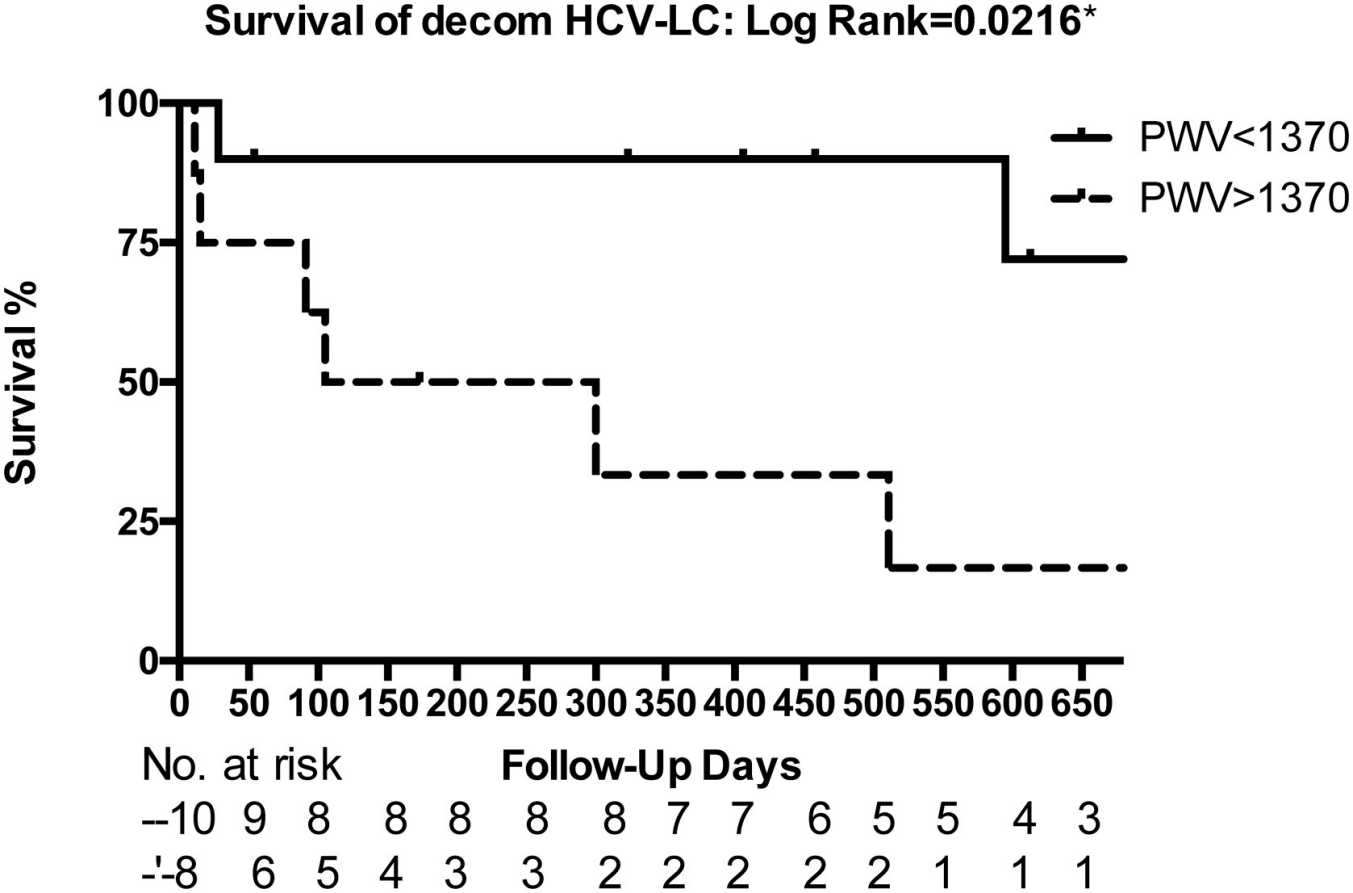
The Kaplan–Meier plot of one-point PWV> or <1370 cm/s predicted mortalities of patients with HCV related decompensated cirrhosis (Log-rank test p = 0.0216). The number of patients at risk on each time point is also shown in the bottom of the figure.

Furthermore, by ROC analysis, One-point PWV predicted mortality only in patients with HCV related decompensated cirrhosis (Fig 1) but neither in hepatitis B nor in alcoholic related liver cirrhosis. Hence Youden’s index was calculated to determine the best cut-off point for them. The result showed that one-point PWV>1370 cm/s could predict mortalities with AUROC = 0.813,p = 0.034. In addition, one-point PWV>1370 cm/s predicted mortalities in HCV related decompensated cirrhosis was further demonstrated by K-M plot (Fig 2, Log-rank test p = 0.0216).

Last, we investigated various non-cardiac and cardiac parameters including systolic, diastolic function, systemic hemodynamic status and CCM for association with overall mortalities in decompensated cirrhosis by multivariable Cox regression analysis.

As shown in Table 4, univariable Cox regression analysis revealed CTP score[29] and One-point PWV>1370 cm/s predicted overall mortalities in decompensated cirrhosis. Multivariable Cox regression analysis also confirmed that only the CTP score and one-point PWV>1370 cm/s were associated with overall mortalities (CTP score: OR 1.762, 95% CI 1.216–2.552, P = 0.003; One-point PWV>1370: OR 6.941, 95% CI 2.004–24.036, P = 0.002) (Table 4) specifically in HCV related liver cirrhosis (AUC = 0.817,p = 0.034).

**Table 4:** Odds ratios for overall mortalities in decompensated cirrhosis in relation to demographic and cardiac variables (Cox regression).

Variables	Crude OR (95%CI)	P value	Adjusted OR (95% CI)	P value
Age	1.044(0.983–1.109)	0.164		
Gender	1.746 (0.394–7.744)	0.463		
**CTP score**	1.718 (1.221–2.417)	**0.002**	1.762(1.216–2.552)	**0.003**
Cr	0.883(0.607–1.283)	0.514		
Na	0.922(0.830–1.025)	0.135		
LVEF	1.009 (0.937–1.085)	0.817		
Absolute GLS	0.879(0.662–1.167)	0.372		
Diastolic dysfunction	1.378(0.489–3.887)	0.544		
CO	1.180(0.892–1.560)	0.246		
**PWV>1370**	5.938(1.808–19.501)	**0.003**	6.941(2.004–24.036)	**0.002**
AC	0.314(0.072–1.370)	0.123		
AI	0.982(0.934–1.033)	0.483		
CCM	1.485(0.527–4.185)	0.454		

CTP score: Child-Turcotte-Pugh score; LVEF: Left ventricular ejection fraction; Absolute GLS: Absolute global longitudinal strain; CO: Cardiac output; PWV: One point pulse wave velocity; AC: carotid arterial compliance; AI: carotid augmentation index; CCM: Cirrhotic cardiomyopathy

## Discussion

CCM is a clinical syndrome in patients with liver cirrhosis and is characterized by an abnormal and blunted response to physiologic, pathologic, or pharmacologic stress and normal-to-increased cardiac output and contractility at rest [30]. In this clinical observational research, by measuring absolute global longitudinal strain, common carotid artery one-point pulse wave velocity (one-point PWV) and various parameters without stress test, we demonstrated that 27.8% cirrhotic patients presented with normal systolic but abnormal diastolic functions and QTc prolongation that were compatible with CCM criteria[16]. 34.2% cirrhotic patients presented with diastolic dysfunction in a resting state compared to 24.1% in control group, although without statistical difference. Systolic functions did not show conspicuous difference between liver cirrhosis and control group or between compensated and decompensated cirrhosis. Nevertheless, the electrophysiological parameter QTc values significantly increased in liver cirrhosis compared to in control group and raised in decompensated cirrhosis compared to in compensated cirrhosis. CO and AC were also significantly higher in cirrhotic patient s than in control group. Most importantly, mean one-point PWV was significantly higher in liver cirrhosis than in control group and higher in CCM than in non-CCM patients. One-point PWV predicted CCM and diastolic dysfunction in liver cirrhosis. Furthermore, although one-point PWV was non-significantly higher in compensated liver cirrhosis, its value > 1370cm/s predicted overall mortalities in decompensated cirrhosis (multivariable Cox analysis OR = 6.941) in addition to CTP score specifically in HCV cirrhosis (AUC = 0.817, p = 0.034).

Moon Young Kim et al. revealed that the baseline EF was significantly higher in patients with a blunted dobutamine stress echocardiography (DSE) response than that of those with a normal DSE response[31]. Yet Kazankov K et al. showed that both systolic and diastolic myocardial functions were compromised in the patients at rest[32]. Karagiannakis et al., used tissue Doppler echocardiography at rest and after a dobutamine stress test to evaluate 45 cirrhotic patients[33]. None had systolic dysfunction, although 17/45 (37.8%) had diastolic dysfunction, as determined by E/e average ratio. In our study as shown in Table 1, systolic functions and absolute speckle-tracking derived strain (GLS) were slightly higher in cirrhotic patients than in control group, although without statistic significance, which corresponded to Karagiannakis’s study[33]. In addition, by formula, cardiac index is in positive correlation to C.O. In Table 1, we also found the C.O. increased from control group to liver cirrhosis and from compensated to decompensated. What we observed tallied with Natig Gassanov’s review that increased cardiac output due to hyperdynamic circulation is a pathophysiological hallmark of CCM[16]. The percentage of CCM in liver cirrhosis (27.8%) in our study was also similar to that found in the dobutamine stress test (25.4%) and not related to compensated or decompensated cirrhosis [31].

To explain the different results in systolic function, Kazankov et al., used tissue Doppler imaging to detect latent cardiac failure at rest in 44 cirrhotic patients[32]. They found that systolic and diastolic myocardial functions were compromised in these patients at rest [32]. Mean peak systolic tissue velocity and mean systolic strain rate were also diminished in these cirrhosis patients, and these findings were not related to the cause of cirrhosis[32]. The results in present study regarding systolic and diastolic dysfunction differ from theirs, probably because of differences in ethnicity (Taiwan and Greece respectively) and major liver cirrhosis etiologies (alcohol cirrhosis in ours 35% vs. 73% in theirs respectively). We classified nonalcoholic cirrhosis into HBV- and HCV-related cirrhosis while Kazankov et al. broadly included a single nonalcoholic cirrhosis group. The different exclusion criteria used in the present study, including the exclusion of patients with DM, HTN, ESRD, ongoing infection, and unstable vital signs to avoid pre-existing cardiovascular disorders, might also explain differences in the present and past results.

Regarding diastolic function, Torregrosa reported worse diastolic function and abnormal systolic response limiting exercise capacity only during stress [34]. Basal diastolic function was similar, while cardiac index and ejection fraction were higher in cirrhotic patients than in healthy controls [34]. Somani PO et al., found that 30% of cirrhotic patients had mild diastolic dysfunction and that there was no difference in relation to liver cirrhosis etiologies or in compensated vs. decompensated cirrhosis [35] in resting state. Kazankov K et al. reported 54% had diastolic dysfunction by Garcia MJ’s methodology[32, 36]. In our cohort study, 34.2% of cirrhotic patients presented with diastolic dysfunction in resting state by the Montreal 2005 consensus criteria, which was similar to what Somani PO et al. found who adopted recommendation issued from the American Society of Echocardiography 2009.

AC increased from control group to cirrhosis and from compensated to decompensated cirrhosis, which confirms the results of a previous study that higher AC in cirrhotic patients as compared with control group [37]. AC is an important clinical variable in cardiovascular disease [38]. Carotid arterial compliance (AC) was positively correlated with circulatory hyperdynamic status, such as plasma volume [37] and increased CO, as demonstrated in the past[39] and present study (R = 0.301, p = 0.019).

In concordance with previous studies [2, 4], we observed increasing resting LA dimensions from compensated to decompensated cirrhosis (Table 1). LVEDD was also higher in decompensated patients to either normal or compensated liver cirrhosis, as noticed in earlier studies [40]. QTc interval prolongation was seen in 63% of the present cirrhotic patients and worsened in decompensated cirrhosis, as in previous studies[2].

Damage to large arteries is an important contributing factor to elevated cardiovascular morbidity and mortality in patients with cardiovascular risk factors, like hypertension [41]. Quantitative information on large arteries, such as arterial distensibility and stiffness, may be obtained indirectly by determining PWV[41]. A PWV value >1300 cm/s, is a strong independent predictor of cardiovascular mortality with high performance values in hypertensive patients [42]. In addition, PWV has been thoroughly studied as a risk predictor for coronary artery disease [43] and as a marker of atherosclerotic vascular damage[44] and all-cause mortality in general populations [45]. Atherosclerosis was also reported to be associated with liver cirrhosis [46] and chronic hepatitis C[47]. The mechanism underlying such associations might be related to vascular wall inflammation caused by monocytes and macrophages. In our study, we used one-point measurement of PWV that was a newer and valid method[48] and highly correlated with carotid-femoral PWV[20]. Moreover, a local measurement of PWV is more precise evaluation of artery condition than regional assessment, taking into account the difference in the structures of arteries between two sites[12].

The mean one-point PWV was significantly higher in liver cirrhosis than in control group and higher in CCM than in non-CCM patients (Table 3). One-point PWV predicted CCM and diastolic dysfunction in liver cirrhosis. Most importantly, one-point PWV value > 1370cm/s predicted overall mortalities in decompensated cirrhosis (multivariable Cox analysis OR = 6.941,p = 0.002) in addition to CTP score, specifically in decompensated HCV related cirrhotic patients (AUC = 0.817, p = 0.034). Subgroup analysis revealed neither hepatitis B nor alcoholic cirrhotic patients’one-point PWV correlated with survival. The percentage of CCM and mortality rate was higher in hepatitis C liver cirrhosis than in hepatitis B liver cirrhosis (Table 2), which corresponded to the important observation studies by Wu VC et al.[49–51]. As we knew, patients infected with HCV have high prevalence of steatosis[52]. HCV infections are associated with hepatic steatosis, type 2 diabetes mellitus, insulin resistance and cardiovascular related diseases (CVD)[53]. Tomiyama H et al., also reported hepatitis C virus but not hepatitis B virus was associated with increased pulse wave velocity[54]. Furthermore, a meta-analysis study demonstrated HCV-infection associated with increased cardiovascular mortalities[50]. The pathophysiology might be related to different T cell response between HBV and HCV[55]. Hepatitis C virus is more resistant to cytokines and to acquire the capacity to survive within a host environment apparently unfavorable to its persistence due to its efficiency in escaping immune surveillance. HCV was also found to be associated with an increased risk of carotid-artery plaque and carotid intima-media thickening[47].

Recently Novo et al.[56] had an important finding that HCV-related cirrhotic patients have increased arterial stiffness as compared to controls. They also observed that the HCV-cirrhotic patients with varices had higher absolute global longitudinal strain (GLS) levels as compared to those without. A trend towards improvement of cardiac function parameters (TAPSE and E’ Lateral) after direct antiviral agents (DAAs) was also demonstrated. Since HCV cause metabolic disorders[57] and DAA can effectively eradicate them, these findings correspond to our findings that only in decompensated HCV-liver cirrhotic patients, carotid one-point PWV > 1370cm/s predicted overall mortalities and their absolute GLS had a trend higher than that in control group (HCV: 22.5, (21.2~23.6) vs. control group: 20.2 (19.1~23.0), M-W U test p = 0.06). They concluded that HCV-related cirrhotic patients should be considered as patients with increased cardiovascular risk and pulse wave velocity seem to be useful and non-invasive method for early detection of vascular damage in HCV-cirrhotic patients[56]. These findings support our highlight on the importance of one-point PWV on mortality of decompensated HCV- cirrhotic patients.

Several limitations of our study should be described. First, we excluded patients with hypertension or hypotension at enrollment, which would exclude those cirrhotic patients with low systolic and/or diastolic blood pressure. The reason for the exclusion was to eliminate a pre-existing cardiovascular disease that could confounded the assessment of CCM-related cardiovascular abnormalities[58] and overall mortalities. Moreover, our study group number was small and included different etiologies of cirrhosis. The relative small number of subjects due to strict inclusion and exclusion criteria was a limitation. Nevertheless, we revealed that no local PWV difference between different etiologies of cirrhosis although the odds ratio for predicting overall survival in decompensated HCV-related liver cirrhosis was high (OR 6.914). The heterogeneity of the study group would hence not influence the results of these relatively stable cirrhotic patients.

## Conclusions

In patients with cirrhosis, 27.8% were diagnosed with CCM by resting cardiovascular parameters. One-point PWV increased in CCM, correlated with diastolic dysfunction. Its value > 1370cm/s predicted overall mortalities in patients with HCV related decompensated cirrhosis (multivariable Cox analysis OR = 6.941,p = 0.002) in addition to CTP score. Further study may be needed to confirm its capability for assessing cardiovascular and mortality risks in HCV related decompensated cirrhosis.

## Supporting information

S1 Fig Absolute global longitudinal strain values from 29 control group participants and 80 cirrhotic patients (31 in compensated group, and 49 in the decompensated group) were compared, as detailed in Table 1. GLS was defined as the mean strain of 18 segments. An abnormal absolute GLS value was defined as ≤ 18% in this study.
(TIFF)

S2 Fig Arterial resistances, including carotid AC and AI, were measured by vascular ultrasonography. . Data obtained from 29 control group participants and 80 cirrhotic patients were compared, as detailed in Table 1.
(TIFF)

S3 Fig One-point PWV also correlated with diastolic dysfunction (R = 0.460, P<0.001, S3 Fig) and able to predict it (OR 1.003, 95% CI 1.001–1.005, P<0.001) by univariable logistic regression analysis. (TIFF)
