# Supplementary figures and images for: In HCV-related liver cirrhosis, local pulse wave velocity increases and in decompensated patients correlates with poorer survival
Source: PLoS One. 2019 Mar 19;14(3):e0212770. doi: 10.1371/journal.pone.0212770 (PMC6424395; doi:10.1371/journal.pone.0212770)

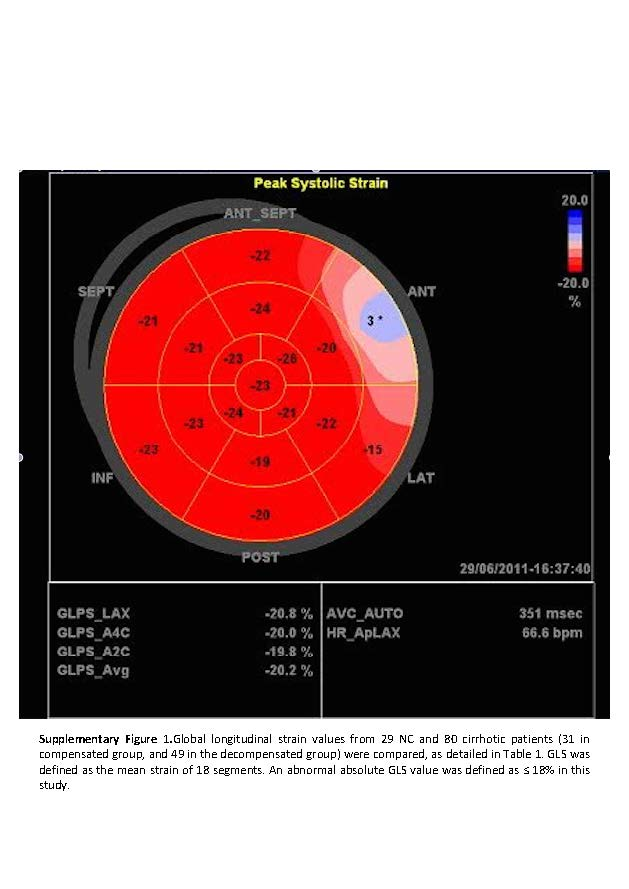

Supplement: S1 Fig — GLS was defined as the mean strain of 18 segments. An abnormal absolute GLS value was defined as ≤ 18% in this study. (TIFF) [file pone.0212770.s001.tiff]

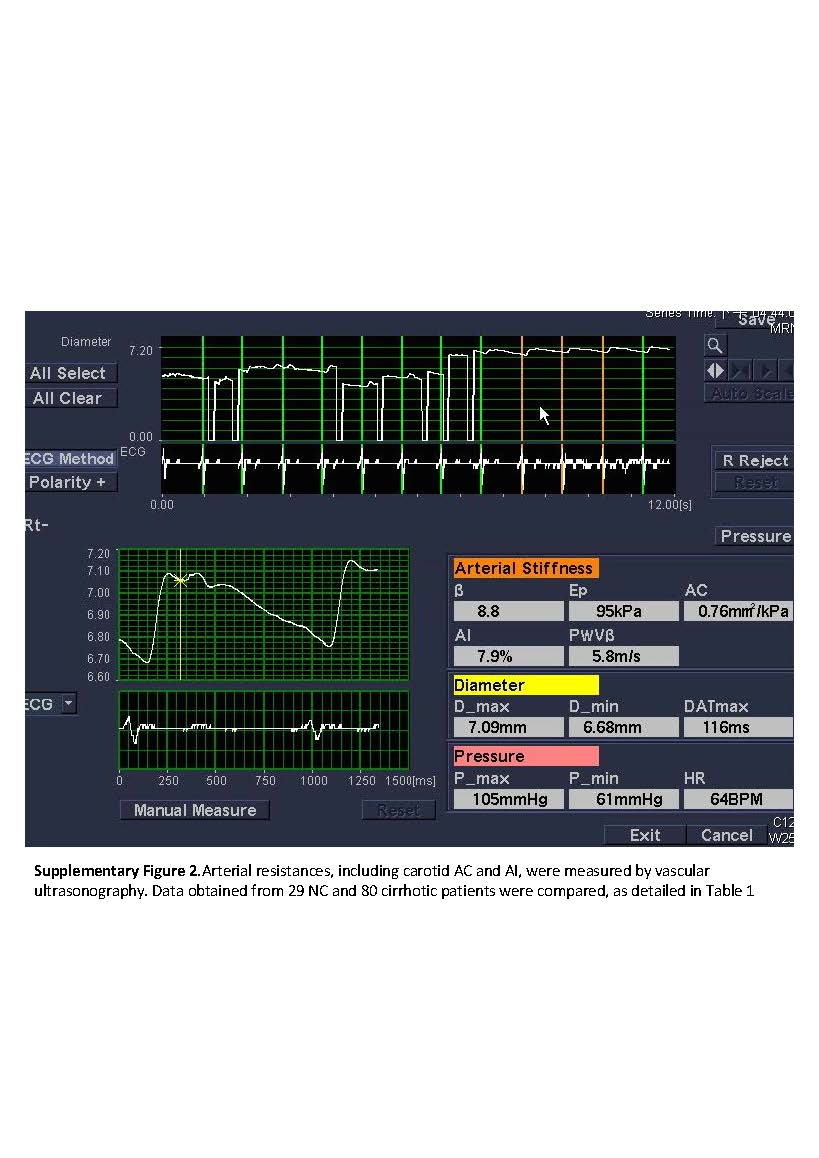

Supplement: S2 Fig — . Data obtained from 29 control group participants and 80 cirrhotic patients were compared, as detailed in Table 1. (TIFF) [file pone.0212770.s002.tiff]
